# Neuronal Calcium Signaling and Cytoskeletal Dynamics in Neurodegeneration

**DOI:** 10.3390/ijms27062550

**Published:** 2026-03-10

**Authors:** Anastasiya Rakovskaya, Ekaterina Volkova, Ekaterina Pchitskaya

**Affiliations:** 1 Laboratory of Biomedical Imaging and Data Analysis, Institute of Biomedical Systems and Biotechnology, Peter the Great St. Petersburg Polytechnic University, Khlopina St. 11, 194021 St. Petersburg, Russia; 2 Laboratory of Molecular Neurodegeneration, Institute of Biomedical Systems and Biotechnology, Peter the Great St. Petersburg Polytechnic University, Khlopina St. 11, 194021 St. Petersburg, Russia

**Keywords:** cytoskeleton neurodegeneration, dynamic tubulin microtubules, spine apparatus, dendritic spines, Alzheimer’s disease

## Abstract

Neuronal function relies on the precise coordination between intracellular calcium (Ca^2+^) signaling and the cytoskeletal architecture that underpins synaptic transmission, plasticity, and structural stability. Disruption of this calcium–cytoskeleton interplay has been noted in numerous neurodegenerative diseases. We discuss how Ca^2+^-dependent cytoskeletal remodeling governs long-term potentiation and depression, dendritic spine morphology, and presynaptic function, highlighting the functions of end-binding proteins, STIM (Stromal Interaction Molecule)/Orai-mediated store-operated calcium entry, and the spine apparatus. Disease-specific manifestations of cytoskeletal–calcium dysregulation are reviewed across Alzheimer’s disease, Parkinson’s disease, amyotrophic lateral sclerosis, tauopathies, and prion disorders. Finally, we evaluate emerging therapeutic strategies targeting calcium homeostasis, cytoskeletal dynamics, and their downstream effectors, including multi-target approaches.

## 1. Introduction

Neurodegeneration refers to the progressive loss of function of neurons, often tied to loss of structure [1], including that of the synaptic compartments. Neurodegeneration in humans can cause a variety of devastating symptoms depending on the class of neurons affected and often leads to fatal outcomes. Some widely studied neurodegenerative diseases include Alzheimer’s disease (AD), Parkinson’s disease (PD), Huntington’s disease (HD), and Amyotrophic Lateral Sclerosis (ALS). These, and other neurodegenerative diseases, exhibit a broad range of clinical symptoms while sharing several common pathological features on the cellular level [2,3,4,5]. Such features include toxic aggregation of proteins [2,4,6], inflammatory responses [3], impaired calcium homeostasis, including endoplasmic reticulum (ER) calcium homeostasis [7,8], oxidative stress [9] and cytoskeletal dysregulation [5,10,11]. The components of the molecular pathways that constitute these features interact with each other, and the study of such interactions is of great interest. A notable example is the influence of intracellular calcium signaling disruption on neuronal cytoskeleton and vice versa. This is especially intriguing for dendritic spines, which govern both synaptic excitatory and inhibitory transmission, with their density and distribution contributing to synaptic function [12,13]. Actin is a primary component of dendritic spines, making actin dynamics critical for their structural plasticity [14]. Increasingly, neurodegenerative diseases are linked to defects in synaptic plasticity, or the ability of synapses to reconfigure over time in response to external cues [15]. Diseases in which synaptic plasticity and associated postsynaptic structure defects are pronounced include AD, PD, HD, schizophrenia (SCZ), and autism spectrum disorder (ASD) [14].

Neurodegenerative disorders have been in the spotlight of countless studies for many years. However, little progress has been made in effective therapy development for most of them. Cytoskeletal dysregulation, including microtubule dysregulation, is a common feature of neurodegenerative and psychiatric disorders [16,17,18,19,20]. Despite that, microtubules are not considered as potential therapeutic targets often enough. In this review, we summarise the findings in the area of cytoskeletal interactions with intracellular calcium signaling in the context of neurodegeneration with additional attention to microtubules, their dynamics and associated proteins. A deeper understanding of this topic is necessary for creating new therapeutic agents and finding new targets to slow down the progression of neurodegeneration.

## 2. The Neuronal Cytoskeleton

Neurons are highly functionally polarised and asymmetric, and their cytoskeleton structure reflects this asymmetry in the vast difference in its structure in different compartments. The cytoskeleton has three major components: actin, microtubules, and neurofilaments. Each is unique, associating with its own set of binding and regulatory proteins and performing specialized roles. Most of the cytoskeletal proteins are synthesized in the neuronal soma and are transported along the axon and dendrites.

### 2.1. The Actin Cytoskeleton

Actin rings, joined by spectrin along with several other proteins, such as adducin, form the membrane-associated periodic skeleton (MPS) found in axons, dendrites and the neuronal soma [21,22]. The MPS is usually considered to be immobile, stable, and resistant to actin and microtubule depolymerization, although its stability has been questioned in some recent studies [23,24]. Several studies have highlighted a broad range of involvements for the MPS in neuronal processes, like stabilization of axons [25,26], electrical conductivity [27,28], lattice-like organization of proteins [29,30], and control of axonal diameter [27]. In addition to the MPS, actin forms other structures in axons, such as hot spots [31], trails [32], actin patches [33,34] and longitudinal actin filament bundles (AFB) [31,35,36]. Actin is also highly concentrated at synapses, and it was recently shown that axonal actin patches are localized to presynaptic sites and that F-actin accumulations become even more prominent when the neuron matures [21]. Actin ultrastructure in the presynaptic bouton comprises an intricate combined meshwork of short, branched and bundled filaments [37]. It is thought that in the presynapses the actin network is mainly Arp2/3-dependent [37], and actin dynamics, mediated by the Arp2/3 complex, have been shown to be critical for synapse formation in flies [38]. Presynaptic actin forms a mesh around synaptic vesicles to act as a scaffold for synaptic vesicle regulators, such as synapsin and bassoon [39,40,41] and acts as a scaffold for protein complexes [42] and the presynaptic boutons in general [39,43]. It also plays a role in endocytosis [44,45] and the transport of recycled vesicles to the synaptic vesicle cluster [46,47]. The presynaptic positioning of cytoskeletal meshwork in nonrelease domains also suggests that actin bands help to restrict synaptic vesicles to release domains [41,48]. Synaptic actin becomes even more enriched upon synaptic activity, suggesting additional recruitment and polymerization of actin at the presynapse [39]. Although the overall thinking regarding the actin involvement in the synaptic vesicle (SV) cycle envisions that actin and actin motors would form a range of fixed, distinct structures within a presynaptic compartment, each of which would facilitate a distinct step of the cycle [49,50,51], a recent study proposes an alternative, although not mutually exclusive, possibility in which synapsin-driven SV condensates may act as molecular beacons for locally sequestering actin and directing its polymerization.

### 2.2. Neurofilaments

Neurofilaments (NF) are neuron-specific intermediate filament proteins that constitute a major component of the neuronal cytoskeleton [52,53]. These proteins are composed of several principal subunits: neurofilament light chain (NF-L), neurofilament medium chain (NF-M), neurofilament heavy chain (NF-H), α-internexin and peripherin [54,55]. Additional splice variants exist, but their distributions and significance are not yet clear because they have not been systematically examined. Neurofilaments are mostly present in large myelinated axons, where they provide rigidity, tensile strength, and contribute to the maintenance of cell shape and intracellular transport [56,57]. They also regulate axonal caliber, which is a key determinant of nerve conduction velocity [54,56]. It has been shown that NF assemblies are a part of the synaptic cytoskeleton [58,59]. Some studies suggest that the NF scaffold acts as a docking platform to organize the topography of organelles within different neuronal compartments [60,61,62].

### 2.3. Microtubules

Another crucial component of the cytoskeleton are the microtubules (MTs). Microtubules are dynamic polymer structures, assembled from α/β-tubulin heterodimers. The exact α- and β-tubulin isotypes that are incorporated into the polymer, their post-translational modifications (PTMs) and microtubule-associated proteins (MAPs) determine the functions of the microtubules and are different in the axonal and somatodendritic compartments of the neuronal cell [63]. During neuronal polarization, the first distinct feature of the proximal axon is microtubule bundling: this network arises even before the assembly of major Axon Initial Segment (AIS) proteins [64]. Microtubules also display different orientations in axons and dendrites in mammalian neurons. Neurites emerging from the neuronal soma contain bundles of microtubules whose orientation differs between axonal and dendritic compartments. In axons, microtubules exhibit a uniform polarity with plus ends oriented distally, whereas in dendrites—particularly in proximal regions—microtubules display mixed polarity [65,66,67,68]. This fundamental difference in microtubule organization determines the directionality of transport mediated by microtubule-based motor proteins, supporting unidirectional cargo trafficking in axons and bidirectional transport in dendrites [65,69,70]. This configuration is mainly established and maintained by motor-dependent MT sliding mechanisms [71] and the control of non-centrosomal MT nucleation [72]. Within the axon shaft, microtubules are heavily stabilized and organized in dense, parallel overlapping bundles. They are anchored at the minus end by proteins such as patronin [73,74,75], calmodulin-regulated spectrin-associated protein 2 (CAMSAP2) [76] and the γ-tubulin–augmin complex [77]. Proteins belonging to the family of plus-end tracking proteins (+TIPs) associate with the growing plus ends of microtubules and regulate their dynamics as well as their interactions with other cellular components. The +TIPs family includes end-binding protein 3 (EB3), a neuron-specific member of the EB protein family [78]. EB3 is expressed in neurons, including dendritic spines, and localizes to the polymerizing ends of dynamic neuronal microtubules [79,80,81,82,83]. It is shown to participate in the link between actin cytoskeleton and microtubules, especially important during neurogenesis [84] and dendrite branching regulation through association with scaffolding protein PSD95 [85].

Longitudinally aligned MTs enable the directional transport of organelles, vesicles, other microtubules and cargoes along the axon, mediated by MT-based motor proteins of the kinesin superfamily and cytoplasmic dynein [63,86,87]. In addition, microtubules partially comprise the AIS filter itself. AIS-specific fascicles enriched in GTP islands and the associated proteins like the tripartite motif-containing protein 46 (TRIM46) and the microtubule crosslinking factor 1 (MTCL1) are physically linked by EB1 and EB3 proteins to ankyrin-G [64,88]. In turn, ankyrin-G interacts with βIV-spectrin and cortical actin, which provides a strong structural basis for the AIS-dependent filter.

### 2.4. Dendritic Spine Cytoskeleton

As neurons mature, dendrites give rise to small, actin-rich protrusions known as dendritic spines, which serve as the primary sites of excitatory synaptic transmission. These structures display pronounced morphological and molecular plasticity, which is essential for learning and memory processes. Actin filaments are highly enriched within dendritic spines, where they form the principal structural scaffold of the postsynaptic density (PSD) and play central roles in synapse formation, growth, and remodeling. Although microtubules are largely confined to axonal and dendritic shafts, they can transiently invade dendritic spines during synaptic maturation. These highly regulated and activity-dependent microtubule invasions are closely associated with synaptic plasticity [89,90,91]. Microtubule entry into spines correlates with structural remodeling, including spine head protrusion and enlargement [89,90,91,92], and may enable the targeted delivery of postsynaptic cargo. Consistent with this notion, the kinesin motor KIF1A and its cargo synaptotagmin-IV have been shown to be co-transported along microtubules into selected dendritic spines in an activity-dependent manner, thereby supporting local synaptic remodeling [93].

In contrast to neuronal growth cones, where actin nucleation and elongation occur concurrently at the leading edge, these processes are spatially segregated within mature dendritic spines [94]. Arp2/3-dependent actin nucleation, driven by the WAVE regulatory complex and functionally associated with PSD-95, is restricted to the spine core [94,95], whereas actin elongation factors such as VASP and formin-like protein 2 are preferentially localized to spine protrusions and filopodial regions. This spatial compartmentalization, coordinated by postsynaptic scaffolding and adhesion proteins, enables rapid and stimulus-dependent remodeling of the F-actin network in response to synaptic activity.

## 3. Molecular Pathways in Cytoskeleton–Calcium Connection

Calcium signaling in neurons is highly complex and spatially diverse, with tightly regulated calcium dynamics acting as a versatile intracellular messenger that coordinates molecular, structural, and functional processes across distinct neuronal compartments. There is a strong link between the components of the cytoskeleton and calcium signaling, with Ca^2+^ concentrations being able to change the structure of the cytoskeleton and vice versa. A recent study discovered that even the MPS, which is usually considered to be static and stable, undergoes calcium signaling-driven local disassembly and reformation constitutively in axons [24]. However, the most intriguing cytoskeleton–calcium signaling interactions occur in the pre- and postsynaptic compartments.

### 3.1. Long-Term Potentiation and Long-Term Depression

Synaptic activity induces long-term potentiation (LTP) or long-term depression (LTD), both of which are triggered by a rise in postsynaptic intracellular calcium. Distinct patterns of calcium signaling—differing in amplitude and duration—activate different intracellular pathways. These signaling cascades result in corresponding structural and functional changes, including spine enlargement and increased receptor numbers during LTP, or spine shrinkage and receptor removal during LTD, processes that are essential for learning and memory. In a study of glutamate-induced LTP [96], the actin-severing protein cofilin was recruited within seconds of stimulation, followed by actin and remodeling factors including Aip1 and Arp2/3. Within 7–60 min after stimulation, these components returned to baseline levels, allowing actin crosslinking proteins such as drebrin and α-actinin to stabilize the cytoskeleton. After approximately one hour, newly synthesized postsynaptic proteins were incorporated to support sustained spine maturation. Based on these observations, a three-phase model of early LTP-associated spine remodeling was proposed, comprising reorganization, stabilization, and consolidation phases. In addition, myosin IIB motors localized in the spine neck were shown to regulate actin dynamics during the reorganization phase [97]. Similar to mechanisms observed in growth cones, synaptic activity-driven microtubule invasion into dendritic spines plays a critical role in coordinating actin network remodeling during synaptic plasticity [91,93,98,99].

Dendritic spines were long thought to be devoid of microtubules, with actin filaments considered the primary determinants of spine morphology and synaptic plasticity. However, it is now well established that dynamic microtubules can transiently invade dendritic spines in an activity-dependent manner, contributing to spine enlargement [79,89,90,99]. This process is calcium-dependent, as blockade of NMDAR activity or intracellular calcium chelation reduces microtubule entry into spines [79,80,100]. Elevated calcium signaling is also correlated with increased F-actin content, and spines with higher actin density are preferentially targeted by dynamic microtubules [80,100]. While the precise mechanism guiding microtubules from the dendritic shaft into spines remains to be fully elucidated, F-actin appears to play a central role in this targeting process.

In addition to mushroom spines, calcium–cytoskeletal interactions also critically influence other dendritic spine subtypes, including stubby, thin, and filopodia-like spines, which represent distinct morphological and functional states in synaptic circuits (thin, stubby, and filopodia spines are well-characterized morphologies distinguished by actin architecture and head/neck dimensions) [94]. Unlike mushroom spines, thin and filopodial protrusions exhibit more dynamic actin turnover and are associated with high plasticity and spine formation processes, suggesting that Ca^2+^ entry through N-methyl-D-aspartate receptors (NMDAR) and downstream signaling cascades may differentially regulate structural transitions among these subtypes [101]. Indeed, calcium influx via NMDARs triggers actin remodeling and activation of calcium-dependent effectors such as CaM kinase pathways, which are essential for spine maturation and stability. Furthermore, stromal interaction molecule 2 (STIM2)-mediated store-operated Ca^2+^ entry (SOCE) influences spine morphology by contributing to local Ca^2+^ homeostasis and actin dynamics, with evidence suggesting that disruptions of STIM/Orai-dependent Ca^2+^ influx can alter the balance between mature spines and immature filopodial protrusions [102]. Microtubule plus-end binding proteins such as EB3 also play a role in spine development beyond mushroom spines: EB3 entry into spines supports actin network stabilization and contributes to spine growth, whereas loss of EB3 promotes thinner, filopodial morphologies [103].

Microtubule invasion into spines is thought to be mediated in part by interactions between microtubule plus-end tracking proteins, such as EB3, and the actin cytoskeleton [79]. This targeting is tightly regulated through coordinated interactions between the actin network and several adaptor and scaffolding proteins, including p140Cap [100], cortactin [99], and drebrin [104], which collectively facilitate the precise delivery of microtubules to postsynaptic compartments.

### 3.2. Calcium–Cytoskeletal Regulation of Synaptic Proteins and Conduction

Beyond their established roles in long-term potentiation (LTP) and long-term depression (LTD), calcium–cytoskeletal interactions critically regulate the molecular composition of synapses and directly influence synaptic conduction. While previous sections have focused primarily on postsynaptic density (PSD) remodeling, calcium-driven cytoskeletal dynamics affect the broader repertoire of synaptic proteins. At excitatory synapses, actin remodeling governs the stabilization, trafficking, and retention of AMPA and NMDA receptors within the PSD. Activity-dependent Ca^2+^ influx through NMDA receptors activates CaMKII-centered signaling cascades that regulate actin polymerization and receptor anchoring, which is essential for synaptic potentiation and structural plasticity [105,106]. Actin dynamics directly control AMPA receptor trafficking and surface expression, as demonstrated by studies showing that ADF/cofilin-mediated actin remodeling is necessary for AMPAR insertion during LTP [107,108,109]. Disruption of actin organization promotes Ca^2+^-dependent AMPAR internalization and impairs receptor stabilization [110]. Moreover, nanoscale organization of Ca^2+^ signaling domains at excitatory synapses modulates receptor function and plasticity, with alterations linked to neurodegenerative processes [111]. In Alzheimer’s disease and related tauopathies, aberrant Ca^2+^ signaling and cytoskeletal destabilization are associated with synaptic AMPA receptor loss and impaired glutamatergic transmission [109].

Inhibitory synapses are equally dependent on cytoskeletal integrity. Clustering of GABA_A receptors requires the scaffolding protein gephyrin, which anchors them to the cytoskeleton and organizes inhibitory postsynaptic densities [112,113]. Gephyrin nanoscale reorganization determines inhibitory synaptic strength and is dynamically regulated during inhibitory LTP [114,115]. Calcium-dependent signaling pathways, including kinase/phosphatase cascades and calpain activation, modulate gephyrin stability and inhibitory receptor clustering, and their dysregulation contributes to excitation–inhibition imbalance in neurological disorders [112,114]. Alterations in inhibitory synaptic proteins have been implicated in Alzheimer’s disease and related network hyperexcitability states.

Beyond molecular composition, calcium–cytoskeletal coupling directly regulates synaptic conduction. At presynaptic terminals, Ca^2+^ influx through voltage-gated calcium channels (CaV) initiates synaptic vesicle docking and fusion, thereby shaping release probability and short-term plasticity [116,117]. Actin cytoskeleton organization at presynaptic boutons regulates vesicle pool mobilization and recovery, with CaV channel subunits interacting with F-actin to control the readily releasable pool size [118]. In addition, presynaptic calcium channels are physically coupled to cytoskeletal and active zone scaffold proteins, linking structural integrity to neurotransmission efficiency [119]. At the postsynaptic site, Ca^2+^ entry through NMDARs coordinates actin remodeling that stabilizes receptor clusters and maintains synaptic current amplitude [105,107]. Cytoskeletal destabilization reduces receptor retention within the PSD and weakens synaptic responses. Collectively, sustained Ca^2+^ dyshomeostasis and cytoskeletal disorganization, which are hallmarks of neurodegenerative disorders, impair both molecular synaptic architecture and functional signal transmission, ultimately promoting large-scale network dysfunction.

### 3.3. Cytoskeletal Interactions with the Endoplasmic Reticulum

The endoplasmic reticulum serves as an intracellular calcium store, and upon ER Ca^2+^ depletion, calcium sensor proteins of the STIM (stromal interaction molecule)family translocate toward the plasma membrane, where they activate calcium influx through TRPC and Orai channels. This process is known as store-operated calcium entry (SOCE). Previous studies demonstrated that EB3 interacts with the neuronal STIM homolog STIM2 in dendritic spines via a Ser-x-Ile-Pro motif located in the cytosolic domain of STIM2. Disruption of this interaction leads to the loss of mushroom-shaped dendritic spines, a proportional increase in thin spines and filopodium-like protrusions and a reduction in the amplitude of SOCE in hippocampal neurons [120]. Interactions with EB proteins also modulate STIM1 clustering [121] as well as the kinetics and amplitude of STIM1-mediated SOCE [122]. Specifically, downregulation of EB1 or disruption of the interaction between STIM1 and EB proteins promotes enhanced STIM1 clustering and, consequently, potentiation of SOCE [123]. In contrast, STIM2 exhibits an opposite regulatory behavior, underscoring functional divergence between these two homologous proteins. In addition, the STIM1–EB1 protein complex regulates ER motility through the tip-attachment complex (TAC), a mechanism by which ER tubules attach to and move along the growing plus ends of microtubules [124,125]. Recent work further demonstrated that disruption of EB protein binding to STIM2 impairs localization of the spine apparatus, an ER-derived structure, within dendritic spines of hippocampal neurons [123].

### 3.4. The Spine Apparatus

A key structural and functional component linking the actin cytoskeleton to calcium signaling is the spine apparatus (SA), a specialized extension of the smooth endoplasmic reticulum (sER) that is selectively present in a subset of mature dendritic spines. The SA plays a central role in shaping both fast and slow Ca^2+^ dynamics within spines and contributes to multiple signaling pathways, including calcium homeostasis, local protein synthesis, and apoptotic signaling [126]. The volume of the SA critically influences the temporal profile of Ca^2+^ transients, their diffusion into adjacent dendritic compartments, and their coupling to Ca^2+^-dependent signaling cascades. Notably, SA volume positively correlates with total spine volume [127] and spine head size, an association indirectly supported by synaptopodin (SP) depletion studies [127,128], and it can be dynamically increased through NMDAR-dependent activity [126].

Synaptopodin, an actin-associated protein linking the actin network to the ER, is essential for the formation and maintenance of the spine apparatus and serves as a molecular hallmark of SA-positive spines. Loss of the SA due to synaptopodin deficiency leads to impaired long-term potentiation (LTP) in the hippocampal CA1 region and deficits in spatial learning, underscoring the functional importance of this structure in synaptic plasticity [129]. Through its association with the actin cytoskeleton, synaptopodin stabilizes spine architecture while enabling localized intracellular Ca^2+^ signaling. Synaptopodin has also been found to colocalise with Pdlim7 (a member of the PDZ (post synaptic density protein, Drosophila disc large tumor suppressor, zonula occludens-1 protein) and LIM (Lin-11, Islet-1, and Mec-3) domain protein family) on the cisternal organelle, a stack of ER cisternae found at axon initial segments of a subset of neurons [130].

One of the principal mechanisms by which the SA modulates synaptic Ca^2+^ dynamics is calcium-induced calcium release (CICR). During synaptic activity, Ca^2+^ influx through postsynaptic NMDARs triggers the activation of ryanodine receptors (RyRs) located on the SA membrane, leading to the release of additional Ca^2+^ from intracellular stores. In hippocampal CA1 neurons, this RyR-mediated CICR is typically confined to the spine head [126], but under certain conditions—particularly in immature neurons—it can propagate along the dendritic shaft and invade neighboring spines [131]. In developing hippocampal tissue (P8–P17), postsynaptic RyRs in the CA3–CA1 pathway facilitate the spread of Ca^2+^ signals from active synapses to adjacent coactive synapses, thereby lowering the threshold for synaptic plasticity induction [131]. The rapid activation of CICR, occurring within tens of milliseconds, is driven by the diffusion of Ca^2+^ ions from the spine head to RyRs localized near the base of the spine neck. Computational modeling and experimental evidence suggest that RyR activation requires the binding of two Ca^2+^ ions and initiates a directed Ca^2+^ flux from the SA toward the dendritic shaft [132]. Importantly, immunohistochemical studies demonstrate colocalization of synaptopodin and RyRs within the SA, highlighting the close link between the actin cytoskeleton and intracellular Ca^2+^ release machinery [128].

In addition to neuron-intrinsic mechanisms controlling SA structure and Ca^2+^ release, dendritic spines are subject to active regulation by glial cells. Microglia directly influence spine formation and elimination. In the developing cortex, transient microglial contact with dendritic shafts induces filopodial protrusions that later mature into spines via local Ca^2+^ transients and actin accumulation [133]. In the adult hippocampus, microglial motility and contact frequency correlate with spine gain, loss, and stability [134], while after global ischemia, increased microglia–spine interactions and phagocytic engagement accompany spine loss, which can be partially rescued by microglial depletion [135]. Moreover, the spatial distribution of microglia creates local domains of enhanced spine instability [136].

Astrocytes also dynamically regulate spine morphology through structural and signaling interactions. Perisynaptic astrocytic processes rapidly extend and retract around dendritic spines (“tripartite synapse”), with stable, larger spines exhibiting more persistent astrocytic contacts [137]. Astrocytic ephrin-A3 signaling through neuronal EphA4 induces spine retraction and morphological remodeling [138], and three-dimensional reconstructions reveal tighter astroglial ensheathment of thin spines compared to larger spines, suggesting stronger diffusion-based neuron–glia signaling at highly plastic spines [139]. Reviews further support a central role for astrocytes in regulating spine density, maturation, and synaptic strength via contact-dependent and secreted mechanisms [140,141,142].

Importantly, despite robust evidence that glia modulate spine birth, stabilization, and pruning, none of the available studies directly demonstrate glial control of spine apparatus biogenesis or maintenance. Current mechanistic data instead indicate that SA incorporation is governed by intrinsic postsynaptic signaling complexes, including the GPR158–PLCXD2 pathway, which regulates SA abundance and dendritic spine maturation independently of glial input [143]. Thus, while glial regulation of spine structure and Ca^2+^ microenvironment may indirectly influence conditions permissive for SA-positive spine stabilization [133,140,141,142,144,145,146,147], direct glial targeting of SA assembly has not yet been demonstrated and remains an open question.

### 3.5. Neurofilaments in Synapses

NF-L was reported to interact with the glutamatergic GluN1, one of the four subunits of the NMDA receptor [148], which is itself a calcium-conducting channel. A study revealed that NF-L stabilises GluN1 at the membrane by protecting it from ubiquitin-dependent degradation. In agreement with the known role of the NMDAR in regulating dendritic spine morphology, NF-L depletion, which decreases GluN1 level in vivo, also decreases the density and length of dendritic spines. Moreover, NF-L depletion was shown to alter neurotransmission and cause NMDAR hypofunction [56,149].

## 4. Disease-Specific Manifestations

### 4.1. Membrane-Associated Periodic Skeleton

Defects in actin function or dysregulation of synaptic actin are implicated in several disorders, such as Down syndrome and Alzheimer’s disease [150]. Altered synapse development and morphology have been observed in a mouse model for Fragile X syndrome, where altered levels of several actin-regulating proteins were observed [151]. Moreover, mutant α-synuclein, associated with familial Parkinson’s disease, was demonstrated to alter the rate of actin polymerization and to disrupt the actin cytoskeleton in vitro [152]. In Creutzfeldt-Jakob disease, the dysregulation of actin-binding proteins such as cofilin and gelsolin significantly contributes to disease progression by disrupting actin turnover and cytoskeletal reorganization. Additionally, actin-based structures, known as tunneling nanotubes, play crucial roles in prion spread by facilitating cell-to-cell transfer of prions [102]. Another protein that constitutes the MPS, spectrin, is cleaved by calcium-activated proteases including calpain and caspase-3, producing spectrin breakdown products (SBDPs) that may be indicative of necrotic and excitotoxic neuronal injury and death and thus serve as biomarkers of neuronal stress and injury [153]. Elevated SBDPs are observed in both acute conditions like traumatic brain injury and chronic neurodegenerative diseases including Alzheimer’s disease, indicating ongoing calcium-mediated cytoskeletal degradation. At least one hereditary neurological disorder, called spino-cerebellar ataxia type 5 (SCA5), is associated with spectrin mutations with the βIII-spectrin gene (SPTBN2) [153].

### 4.2. Dynamics of Microtubules in Health and Neurodegenerative Diseases

**Figure 1:**
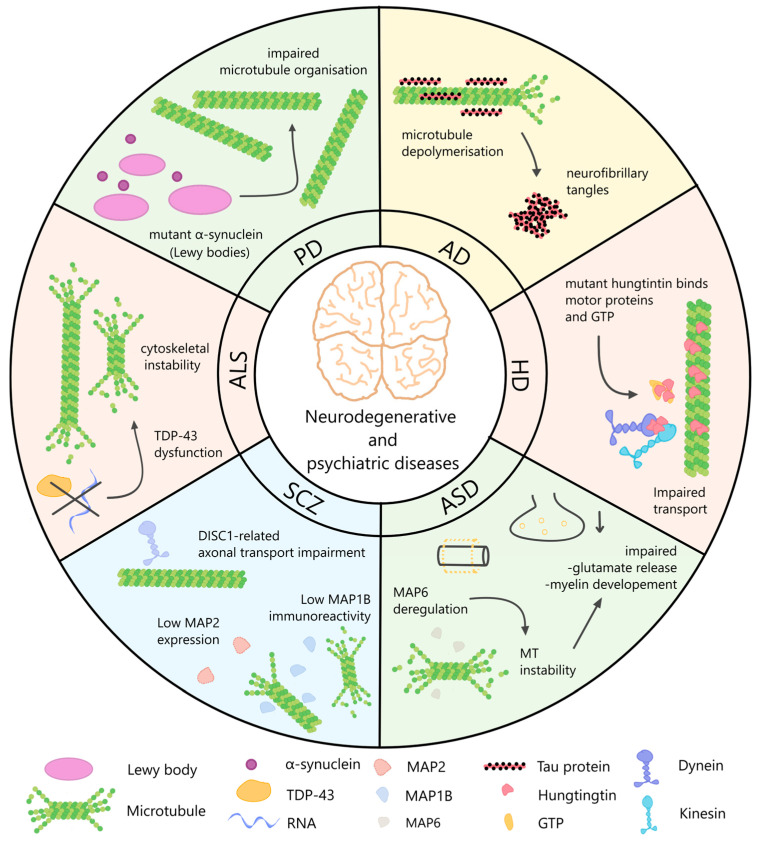
Microtubule dysfunction across neurodegenerative and psychiatric diseases. The schematic summarizes disease-specific alterations in microtubule organization and cytoskeletal regulation associated with major neurodegenerative and psychiatric disorders. In Alzheimer’s disease, tau detachment from microtubules promotes microtubule depolymerization and formation of neurofibrillary tangles. In Parkinson’s disease, mutant alpha-synuclein accumulates in Lewy bodies and disrupts microtubule organization. Huntington’s disease is characterized by mutant huntingtin interactions with motor proteins and GTP, leading to impaired axonal transport. In amyotrophic lateral sclerosis, TDP-43 dysfunction contributes to cytoskeletal instability and microtubule disorganization in motor neurons. Schizophrenia is associated with reduced MAP2 expression and DISC1-related defects in microtubule-dependent axonal transport. In autism spectrum disorder, dysregulation of MAP6 leads to microtubule instability, impaired glutamate release, and altered myelin development.

Precisely regulated microtubule dynamics are critical for axonal and dendritic trafficking, synaptic plasticity, and structural remodeling. Disruption of this delicate balance is increasingly associated with neurodegenerative disease [154,155]. Accumulating evidence from multiple neurodegenerative disorders implicates perturbed microtubule dynamics in disease pathogenesis. Abnormal tubulin post-translational modifications, such as excessive polyglutamylation, directly induce neurodegeneration and impair axonal transport in mouse models, potentially contributing to phenotypes observed in Parkinson’s, Huntington’s, and Alzheimer’s diseases [156]. Altered microtubule dynamics are also a hallmark of tauopathies, in which hyperphosphorylated tau dissociates from microtubules, destabilizing them and impairing transport, ultimately driving progressive neuronal dysfunction and death [157]. Some of the most prominent pathways that lead to microtubule dysfunctions in neurodegenerative and psychiatric diseases are depicted in Figure 1. Similarly, in models of frontotemporal dementia, abnormal microtubule dynamics distort nuclear architecture and disrupt nucleocytoplasmic transport, linking microtubule instability to early pathogenic events [158].

More broadly, disturbances in cytoskeletal homeostasis are observed across Alzheimer’s disease, Parkinson’s disease, amyotrophic lateral sclerosis, Huntington’s disease, and prion disorders, underscoring microtubule dysfunction as a unifying contributor to neurodegeneration [159] (Figure 1). Additional post-translational modifications, including reduced acetylation and increased detyrosination and polyglutamylation, further compromise microtubule stability and motor protein function, with profound consequences for intracellular trafficking and neuronal survival [160].

Currently identified +TIPs proteins, covering the dynamic microtubule plus end, associated with various neuropathological conditions are summarized in [161], but the precise information about their role is still very limited. Beyond the nervous system, dynamic microtubules play central roles in cancer progression, where tumor cells exploit tubulin dynamics to support mitotic spindle assembly, migration, and metastasis. Alterations in tubulin isotype composition and microtubule-associated protein interactions are associated with tumor aggressiveness and therapeutic resistance [162,163,164].

**Figure 2:**
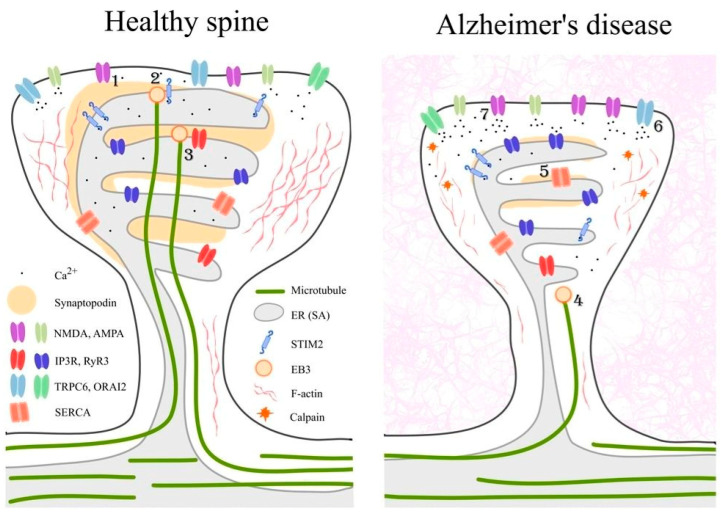
Calcium and cytoskeletal dysregulation in dendritic spines under physiological conditions and in Alzheimer’s disease. A simplified schematic representation of major events occurring in dendritic spines upon physiological condition (**left**) or Aβ oligomer exposure (**right**). A detailed description of pathways is given in the 2nd, 3rd and 4th sections. The schematic illustrates molecular organization of a healthy dendritic spine (**left**) compared with a spine in Alzheimer’s disease (**right**). In healthy spines, coordinated activity of synaptic receptors, calcium channels, and intracellular calcium stores maintains calcium homeostasis and supports actin and microtubule dynamics essential for spine stability and plasticity. In Alzheimer’s disease, disrupted calcium signaling leads to elevated cytosolic calcium, activation of calpain, cytoskeletal destabilization, reduced microtubule invasion, and loss of synaptic proteins. 1—Synaptopodin dependent coupling of NMDA receptors supports calcium signaling and spine stability; 2—STIM2 interaction with the microtubule plus end binding protein EB3 promotes activity dependent microtubule entry into dendritic spines; 3—STIM2 may mediate coupling to IP3 receptors, representing a potential mechanism for regulating calcium release from the spine apparatus endoplasmic reticulum; 4—EB3 associated microtubule invasion exerts neuroprotective effects by stabilizing spine structure and synaptic function; 5—Positive allosteric modulation of SERCA enhances calcium reuptake into the endoplasmic reticulum and limits cytosolic calcium overload; 6—Activation of TRPC6 channels by piperazine derived compounds promotes synaptoprotection and calcium homeostasis; 7—Modulation of NMDA receptor activity contributes to normalization of calcium influx under pathological conditions.

Beyond their roles in cytoskeletal organization and ER dynamics, EB proteins have also emerged as important modulators of neuronal survival under pathological conditions. Overexpression of EB3 exerts a neuroprotective effect in primary hippocampal neurons derived from the PS1-M146V-KI mouse model of Alzheimer’s disease [120], as well as under conditions of amyloid toxicity [165], which is considered a major driver of Alzheimer’s disease pathology. These findings are consistent with emerging evidence that EB family proteins more broadly contribute to neuronal resilience in neurodegenerative conditions. In particular, recent work has shown that EB1 interacts with tau protein, increases the dynamics of tau condensates, and inhibits pathological tau aggregation, thereby delaying the progression toward neurotoxic assemblies in tauopathies [166]. It has also been demonstrated that EB3 binds to inositol 1,4,5-trisphosphate receptors (IP3Rs) through an S/TxIP EB-binding motif in endothelial cells, affecting calcium signaling [167]; however, little is known about this interaction in neurons. Some of the interactions of EB3 and its neuroprotective effects are illustrated in Figure 2. These studies suggest that EB proteins can modulate multiple disease-relevant pathways, including synaptic stability and protein aggregation, positioning them as important cytoskeletal regulators in the context of Alzheimer’s disease.

Together, these findings have stimulated renewed interest in microtubule-targeting agents, including both stabilizers and destabilizers, as potential therapeutic strategies to restore microtubule homeostasis in neurodegenerative and oncologic settings [162,163]. Advances in structural biology and rational drug design continue to identify novel tubulin binding sites and modulators that may selectively fine-tune microtubule dynamics with improved specificity and brain penetrance, further expanding therapeutic opportunities [164]. Collectively, these studies highlight that precise regulation of microtubule dynamics is fundamental for cellular health, and that its disruption represents a central driver of disease processes ranging from synaptic dysfunction and neurodegeneration to cancer.

### 4.3. The Role of the Spine Apparatus in Alzheimer’s Disease Pathology

Importantly, synaptic activity is essential for maintaining the morphology of mature dendritic spines. Blockade of neuronal activity with tetrodotoxin (TTX) induces STIM-dependent spontaneous Ca^2+^ transients and shifts spine populations toward immature filopodial structures [101,168,169]. Dysregulation of STIM/Orai-mediated SOCE has also been implicated in neurodegenerative diseases, particularly Alzheimer’s disease. STIM1 and STIM2 contribute to neuronal Ca^2+^ homeostasis (Figure 2) and are involved in the production of β-amyloid peptide (Aβ). Aberrant expression or activity of these proteins may lead to pathological Ca^2+^ signaling, synaptic dysfunction, and the progression of neurodegenerative processes [170]. In this context, synaptopodin, a key actin-associated protein required for spine apparatus formation and local calcium regulation, has emerged as an important link between calcium homeostasis, synaptic stability, and neurodegeneration. Synaptopodin critically regulates Hebbian synaptic plasticity by controlling actin organization and calcium signaling within dendritic spines, processes that are highly sensitive to STIM-dependent Ca^2+^ dynamics [171]. Clinical and proteomic studies further support a disease-relevant role for synaptopodin, as reduced SP levels have been detected in neuronal-derived exosomes and post-mortem brain tissue from patients with Alzheimer’s disease (Figure 2), frontotemporal dementia, and Lewy body dementias, correlating with synaptic loss and cognitive impairment [172,173]. Importantly, experimental evidence from mouse models demonstrates that synaptopodin dysfunction directly influences disease progression. In the 3xTg model of Alzheimer’s disease, synaptopodin deficiency ameliorates synaptic plasticity deficits and reduces amyloid- and tau-associated pathology, highlighting its functional involvement in disease mechanisms rather than serving merely as a marker of synaptic loss [103]. At the mechanistic level, proximity proteomics has revealed that synaptopodin organizes a specialized molecular network within the spine apparatus that integrates actin remodeling, ER-derived calcium stores, and signaling proteins, positioning SP as a structural and signaling hub whose disruption may exacerbate STIM/Orai-mediated calcium dysregulation in disease [130] (Figure 2). Consistent with this view, alterations in synaptopodin-positive spine densities have also been linked to neuropsychiatric symptoms in aging and dementia, further underscoring the vulnerability of SP-dependent synaptic compartments to pathological calcium signaling.

### 4.4. Neurofilaments in Neurodegeneration

Neurofilaments accumulate abnormally in several major neurodegenerative disorders, including Alzheimer’s disease, Parkinson’s disease, Lewy body disease, and amyotrophic lateral sclerosis [174,175,176]. Mutations in neuronal intermediate filament genes are pathogenic for conditions like Charcot-Marie-Tooth disease and amyotrophic lateral sclerosis [177]. Neurofilament proteins are obligate heteropolymers and contain intrinsically unstructured regions in which most mutations that cause or predispose to disease occur [56,178]. During axon remodeling or pathological circumstances, such as diffuse axonal injury, there is a release of neurofilaments, resulting in neuroinflammation, causing permanent disability in neurodegenerative disorders [53]. The aforementioned release of NF is also the reason why NFs are considered to be great biomarkers for neurodegeneration and injuries [55,56,178]. NF gene mutations are well recognized as causes of several neurological disorders mainly involving degeneration of peripheral nerve fibers [179]. Changes in levels and phosphorylation of NF subunits have consistently been noted in certain psychiatric disorders, although the location of these changes within neurons is poorly understood [53].

All NF proteins are substrates of multiple proteolytic systems; for example, when axoplasm is exposed to the extracellular fluid elevated calcium concentrations activate calpains that readily cleave NFs and promote neurofilament degradation [180,181,182]. Low expression of calcium-binding proteins (parvalbumin, calbindin) in vulnerable neuron populations reduces resilience to transient calcium rises, predisposing to proteolysis and NF pathology in diseases such as ALS [183].

## 5. Therapeutic Strategies and Translational Potential

### 5.1. Targeting Calcium Channels and Transporters

Modulating specific calcium channels represents a direct approach to restoring calcium homeostasis. TRPV4 channel blockade attenuates pyroptosis in epilepsy models and improves alpha-synuclein degradation in Parkinson’s disease models [184,185]. These findings suggest TRPV4 as a therapeutic target across multiple neurological conditions. Store-operated calcium entry modulation shows disease-specific and region-specific effects. Pharmacological inhibition of SOCE may benefit the Huntington’s disease striatum but could be detrimental to cortical and hippocampal neurons [186]. This complexity necessitates careful consideration of brain region and disease context when targeting SOCE pathways. IP3 receptor inhibition with compounds like 2-APB and enoxaparin demonstrates neuroprotective effects by preventing intracellular calcium overload [187]. NMDA receptor antagonists like memantine and L-type voltage-gated calcium channel antagonists like nimodipine have been tested in Alzheimer’s disease, though clinical results have been mixed [188]. The challenge lies in achieving sufficient calcium modulation to provide neuroprotection without disrupting normal calcium-dependent processes essential for neuronal function.

Consistent with this challenge, development of disease-modifying therapies based on the amyloid hypothesis has proven difficult, prompting interest in alternative frameworks such as the calcium hypothesis of neurodegeneration. One intuitive strategy has been direct inhibition of calcium influx or release channels. Indeed, calcium channel inhibitors have demonstrated beneficial effects in animal models of Alzheimer’s disease, Parkinson’s disease, amyotrophic lateral sclerosis, Huntington’s disease, and spinocerebellar ataxia [189,190,191,192,193,194,195,196,197,198]. However, clinical translation has been limited by the essential roles of these channels in peripheral tissues. For example, despite strong preclinical support, ryanodine receptor inhibitors have not advanced to clinical trials in neurodegenerative diseases, largely due to concerns over cardiac dysfunction associated with inhibition of the cardiac RyR2 isoform.

The largest clinical effort targeting calcium channels was the phase III STEADY-PD III trial of the neuronal CaV1.3 L-type calcium channel inhibitor isradipine in Parkinson’s disease. Despite compelling preclinical rationale [199], the trial failed to meet its primary and secondary endpoints [200]. Post hoc analyses suggested that greater drug exposure delayed initiation of dopaminergic therapy, implying that insufficient dosing—limited by peripheral blood pressure effects—may have contributed to trial failure [199]. This outcome further illustrates how peripheral calcium channel functions can constrain effective central nervous system targeting.

Beyond ion channels, calcium-dependent effectors such as the phosphatase calcineurin (CaN) have attracted significant attention. CaN overactivation, driven by elevated cytosolic calcium, contributes to synaptic loss and impaired autophagy in Alzheimer’s disease neurons [193,198,201]. Inhibition of CaN has shown beneficial effects in animal models of brain aging, and epidemiological analyses reveal reduced Alzheimer’s disease incidence among transplant patients chronically treated with the CaN inhibitor FK-506 [202]. Nevertheless, clinical application is limited by the essential role of CaN in immune activation, as CaN inhibitors function as potent immunosuppressants [203].

In addition to broadly targeting calcium channels, increasing attention has been directed toward selective modulation of neuronal calcium-permeable channels as a means to achieve synaptoprotection while minimizing peripheral side effects. One such promising target is TRPC6 (Transient Receptor Potential Canonical 6), a calcium-permeable channel implicated in synaptic stability and plasticity [204,205]. Recent studies have demonstrated that pharmacological activation of TRPC6 can confer neuroprotective and synaptoprotective effects in Alzheimer’s disease models [204]. In particular, two novel small molecules, a piperazine-derived compound (cmp2) [206,207] and a benzopyran derivative (C20) [208], have been evaluated in cellular and animal models of Alzheimer’s disease. Notably, cmp2 selectively activated TRPC6 channels without affecting closely related TRPC3 or TRPC7 isoforms. Both compounds robustly restored dendritic spine morphology under conditions of amyloid-induced toxicity in vitro, indicating pronounced synaptoprotective activity. Furthermore, in transgenic 5xFAD mice, treatment with either compound led to significant improvements in cognitive performance in behavioral assays, while cmp2 additionally ameliorated motor deficits.

An alternative strategy to stabilize neuronal calcium signaling avoids blocking calcium entry or downstream effectors and instead enhances calcium extrusion from the cytoplasm. Pharmacological stimulation of calcium pumps, particularly the sarco/endoplasmic reticulum Ca^2+^-ATPase (SERCA), has emerged as a promising approach. Positive allosteric modulators (PAMs) of SERCA were identified through high-throughput screening targeting SERCA–phospholamban interactions, leading to the development of compounds such as CDN1163 [209]. These lipophilic molecules accelerate the SERCA pumping cycle without altering calcium affinity, facilitating calcium clearance while minimizing basal side effects [210].

SERCA PAMs have demonstrated neuroprotective effects across multiple disease models. CDN1163 ameliorated dyskinesia in a rat Parkinson’s disease model, exerted neuroprotection in Drosophila Parkinson’s disease models, and improved hippocampal memory in APP/PS1 mouse models of Alzheimer’s disease [211,212,213]. Newer compounds, including NDC-1173 and NDC-9009, further enhanced cognitive performance and synaptic integrity in Alzheimer’s disease models [214,215]. Notably, NDC-9009 restored long-term potentiation deficits, normalized aberrant hippocampal network activity observed via miniscope imaging, rescued memory impairments, and reduced amyloid plaque burden in 5xFAD mice [216].

### 5.2. Modulating Cytoskeletal Dynamics

Modulation of cytoskeletal dynamics represents a complementary therapeutic strategy with significant translational potential for neurodegenerative diseases. The neuronal cytoskeleton, primarily composed of microtubules and actin filaments, is essential for maintaining axonal transport, synaptic integrity, and overall neuronal architecture. Dysregulation of cytoskeletal organization is a common pathological hallmark across neurodegenerative disorders, making these structures attractive pharmacological targets.

Pharmacological modulation of microtubule dynamics is well established in oncology and provides a strong proof of principle for targeting the cytoskeleton with small molecules. Microtubule stabilizers, such as taxanes, and microtubule destabilizers, including vinca alkaloids, have been approved and widely used as chemotherapeutic agents for several decades [217,218]. At submicromolar concentrations, taxanes such as paclitaxel suppress microtubule catastrophe events and increase intracellular microtubule mass, whereas vinca alkaloids like vinblastine primarily inhibit the rates of microtubule polymerization and depolymerization. In rapidly dividing cancer cells, suppression of microtubule dynamics prevents progression from metaphase to anaphase, ultimately triggering apoptotic cell death [219,220]. Importantly, these findings demonstrate that microtubule dynamics can be precisely regulated pharmacologically, encouraging adaptation of this approach for neurodegenerative conditions, where the therapeutic goal is cytoskeletal stabilization rather than cytotoxicity.

In the context of neurodegeneration, microtubule-stabilizing strategies remain an active area of preclinical investigation. These studies include development of brain-penetrant epothilone and taxane analogs, as well as identification of novel polypharmacological compounds designed to restore tau–microtubule interactions. One such compound, the PHOX derivative PHOX15, has been shown to restore physiological interactions between aggregation-prone tau and microtubules while simultaneously inhibiting tau kinases [221]. Quantitative analyses demonstrated that PHOX15 reduced early tau aggregation steps in vitro and normalized tau–microtubule binding in neuronal models, as assessed using live-cell tauopathy imaging and aggregation assays [222]. In parallel, modulation of actin cytoskeleton dynamics has emerged as another promising avenue. Actin remodeling, mediated through actin-binding proteins and associated signaling pathways, is critical for synaptic plasticity and spine morphology and has shown therapeutic potential in Creutzfeldt–Jakob disease and related disorders [102].

Several intracellular signaling pathways that regulate cytoskeletal organization have also become key therapeutic targets. Inhibition of Rho-associated kinase (ROCK) and glycogen synthase kinase 3β (GSK-3β) aims to restore cytoskeletal stability while simultaneously suppressing pathological signaling [223]. Notably, dual inhibition of GSK-3β and DYRK1A reduces tau hyperphosphorylation and ameliorates disease phenotypes in Alzheimer’s disease models [224]. Similarly, histone deacetylase 6 (HDAC6) inhibitors, such as MPT0G211, improve microtubule acetylation and stability, leading to reduced tau phosphorylation and cognitive deficits in preclinical studies [224].

Upstream modulators such as Fyn kinase further expand the therapeutic landscape. Inhibition of Fyn reduces protein aggregation, increases synaptic density, and improves memory performance in tauopathy models [224]. Importantly, many of these kinases regulate both cytoskeletal dynamics and calcium-dependent signaling pathways, highlighting a mechanistic convergence between calcium dysregulation and structural neuronal degeneration.

### 5.3. Calpain Inhibition and Proteostasis

Calpain inhibition represents a direct and mechanistically grounded strategy for preventing calcium-mediated cytoskeletal degradation and proteostatic failure in neurodegenerative diseases. Calpains are calcium-activated cysteine proteases that, under physiological conditions, participate in synaptic remodeling and signal transduction. However, sustained intracellular calcium elevation leads to pathological calpain overactivation, resulting in cleavage of cytoskeletal proteins, synaptic components, and key regulators of protein homeostasis. This positions calpains as critical mediators linking calcium dyshomeostasis to structural neuronal damage and aggregation-prone protein pathology.

Early genetic studies provided compelling evidence for this role. Overexpression of the endogenous calpain inhibitor calpastatin markedly reduced amyloid plaque burden, prevented tau hyperphosphorylation, and preserved synaptic integrity in transgenic Alzheimer’s disease models [153]. These dramatic effects established calpain activation as a central downstream effector of calcium toxicity rather than a secondary bystander. More recent work has reinforced this concept by demonstrating that calpain overactivation contributes to synaptic loss, axonal degeneration, and impaired autophagic flux across multiple neurodegenerative conditions, including Alzheimer’s disease, Parkinson’s disease, amyotrophic lateral sclerosis, and Huntington’s disease [225,226].

Calpain-mediated cleavage of tau, α-synuclein, and TDP-43 has been shown to generate aggregation-prone fragments that accelerate pathological seeding and propagation [227,228]. In parallel, calpain activity has been linked to impairment of the autophagy–lysosomal pathway through proteolysis of key regulators such as Beclin-1 and Atg proteins, thereby exacerbating intracellular protein accumulation [229]. These findings position calpains at the intersection of calcium dysregulation, cytoskeletal destabilization, and defective protein clearance.

Pharmacological calpain inhibition has shown encouraging results in preclinical models, including reduced synaptic degeneration, improved behavioral outcomes, and attenuation of protein aggregation [230]. Nevertheless, translation to the clinic remains challenging. Major obstacles include achieving sufficient brain penetration, avoiding off-target inhibition of other cysteine proteases, and preserving physiological calpain functions essential for synaptic plasticity. These limitations have prompted renewed interest in indirect strategies that limit pathological calpain activation without complete enzymatic blockade.

One such approach involves targeting upstream calcium entry pathways that drive calpain overactivation. Recent studies have identified channels such as TRPV4 as contributors to sustained calcium influx in disease models, and pharmacological TRPV4 inhibition attenuates calpain activation, reduces cytoskeletal damage, and improves proteostasis in models of epilepsy and Parkinson’s disease [184,185]. This strategy offers the advantage of dampening pathological calcium signals while preserving basal calpain activity.

### 5.4. Multi-Target Approaches

Given the interconnected nature of cytoskeletal and calcium dysfunction, multi-target therapeutic approaches may prove most effective. Tau-targeting strategies include inhibiting tau aggregation (e.g., OLX-07010, hydromethylthionine mesylate), modulating tau phosphorylation, enhancing tau clearance through autophagy, and immunotherapy approaches like Donanemab [224]. These strategies address tau and amyloid pathology while indirectly affecting calcium homeostasis and mitochondrial function. It is important to note, however, that antibody-based approaches [224] may cause brain hemorrhages and swelling known as amyloid-related imaging abnormalities, which in turn may cause seizures. Multiplexed gene transfer strategies employing silencing and/or over-expressing multiple effectors to preserve vulnerable neurons represent innovative approaches [231]. Modulating tau phosphorylation, enhancing amyloid-beta clearance, regulating glial activation, restoring calcium homeostasis, and preserving mitochondrial function simultaneously may provide synergistic benefits [232]. Emerging therapeutic strategies for neurovascular barrier dysfunction include targeting microRNAs, statins, edaravone dexborneol, minocycline, and stem cell-derived extracellular vesicles to stabilize tight junctions and protect the blood–brain barrier [233]. These approaches address the vascular component of neurodegeneration while potentially affecting neuronal calcium and cytoskeletal function.

## 6. Critical Gaps and Future Directions

Despite substantial progress, significant gaps remain in understanding cytoskeleton–calcium interactions in neurological diseases. The temporal sequence of events (whether calcium dysregulation precedes cytoskeletal disruption or vice versa) remains largely unanswered and likely varies by disease and stage. Resolving this question will require approaches capable of capturing early, pre-symptomatic molecular changes rather than relying on endpoint pathology. Longitudinal studies using advanced imaging techniques in animal models and patient-derived systems are needed to establish the causes of pathology and identify optimal intervention windows. Emerging tools such as in vivo two-photon calcium imaging, genetically encoded calcium indicators, super-resolution cytoskeletal reporters, and longitudinal miniscope recordings offer unprecedented opportunities to track calcium–cytoskeleton coupling across disease progression.

The cell-type specificity of cytoskeleton–calcium dysfunction requires further investigation. Neurons, astrocytes, microglia, and oligodendrocytes exhibit distinct calcium signaling mechanisms and cytoskeletal organizations, yet most studies focus on neurons. Understanding glial contributions to pathology and identifying glial-specific therapeutic targets represents an important frontier [234,235,236]. Astrocytic calcium waves, microglial actin remodeling during immune surveillance, and oligodendrocyte cytoskeletal dynamics during myelination may each contribute uniquely to disease initiation and progression. Future studies integrating single-cell transcriptomics, spatial proteomics, and cell-type-specific genetic perturbations will be critical to disentangle these contributions.

Brain region-specific differences in calcium channel expression and cytoskeletal protein composition create challenges for therapeutic development. The observation that SOCE inhibition may benefit the striatum but harm the cortex in Huntington’s disease exemplifies this complexity [186]. Developing region-selective or cell-type-selective interventions may be necessary for optimal therapeutic efficacy. This may involve exploiting regional differences in channel subunit composition, kinase signaling context, or cytoskeletal isoform expression to achieve therapeutic precision. Advanced delivery strategies, including viral vectors, nanoparticle-based targeting, and ligand-directed drug conjugates, may enable such spatial selectivity.

The role of cytoskeleton–calcium interactions in disease propagation, particularly for prion-like spreading of tau and alpha synuclein, requires deeper investigation. Actin-based tunneling nanotubes facilitate prion spread [102], and extracellular vesicles with altered proteomic profiles may propagate dysfunction [237]. Understanding these intercellular mechanisms could reveal novel therapeutic targets. Future work should determine how calcium-dependent cytoskeletal remodeling regulates vesicle trafficking, nanotube formation, and synaptic vulnerability during pathological protein transmission. Disrupting these processes may allow selective blockade of disease spread without impairing normal synaptic communication.

Translating preclinical findings to clinical success remains challenging. Many compounds showing promise in animal models have failed in clinical trials, possibly due to species differences, inadequate target engagement, or intervention at late disease stages. Developing better biomarkers of cytoskeletal and calcium dysfunction, including spectrin breakdown products [153], could enable earlier diagnosis and treatment monitoring. Additional candidate biomarkers include phosphorylated cytoskeletal fragments, calcium-regulated protease products, and imaging-based readouts of synaptic and axonal integrity. Integrating fluid biomarkers with functional imaging may provide multidimensional measures of target engagement.

The potential for personalized medicine approaches based on patient-specific iPSC models deserves exploration. Given the heterogeneity in tau domain exposure [238] and genetic backgrounds affecting calcium signaling [239], patient-stratified therapeutic strategies may improve outcomes. High-throughput screening using patient iPSCs could identify individuals most likely to respond to specific interventions. Coupling iPSC-based phenotyping with genome-wide association data and machine-learning approaches may further refine patient stratification. Ultimately, integrating cytoskeletal and calcium signaling profiles into precision medicine frameworks may enable rational matching of patients to mechanism-based therapies.

**Glossary**

**Neurodegenerative Diseases**

- Alzheimer’s disease (AD)

A progressive neurodegenerative disorder characterized by amyloid-β plaques, tau tangles, synaptic loss, and cognitive decline. Strongly associated with calcium dyshomeostasis and cytoskeletal disruption.

- Parkinson’s disease (PD)

A movement disorder marked by dopaminergic neuron degeneration and α-synuclein aggregation (Lewy bodies). Involves altered calcium signaling and microtubule dysfunction.

- Amyotrophic lateral sclerosis (ALS)

A fatal disorder involving progressive motor neuron degeneration, linked to neurofilament pathology, calcium imbalance, and cytoskeletal instability.

- Huntington’s disease (HD)

A genetic neurodegenerative disease caused by mutant huntingtin protein, associated with disrupted axonal transport and calcium dysregulation.

- Tauopathies

Neurodegenerative disorders characterized by pathological aggregation of hyperphosphorylated tau protein.

- Prion Disorders (e.g., Creutzfeldt–Jakob disease)

Fatal neurodegenerative diseases caused by misfolded prion proteins, associated with actin dysregulation and calcium-mediated toxicity.

**Cytoskeletal Components**

- Actin

A filamentous protein forming microfilaments; essential for dendritic spine structure, synaptic plasticity, and spine remodeling.

- F-actin (Filamentous actin)

Polymerized actin forming structural scaffolds in dendritic spines and presynaptic compartments.

- Membrane-Associated Periodic Skeleton (MPS)

A spectrin-actin lattice structure in axons and dendrites that regulates axonal diameter, stability, and electrical properties.

- Microtubules (MTs)

Dynamic polymers composed of α/β-tubulin heterodimers; critical for intracellular transport, neuronal polarity, and synaptic plasticity.

- Microtubule-Associated Proteins (MAPs)

Proteins that bind microtubules and regulate their stability and dynamics (e.g., tau, MAP2).

- Neurofilaments (NF)

Neuron-specific intermediate filaments composed of NF-L, NF-M, and NF-H subunits; regulate axonal caliber and conduction velocity.

- Post-Translational Modifications (PTMs)

Chemical modifications (e.g., acetylation, detyrosination, polyglutamylation) that regulate tubulin and cytoskeletal protein function.

**Dendritic Spine Structures**

- Dendritic Spines

Actin-rich protrusions on dendrites that serve as the primary sites of excitatory synaptic transmission.

- Postsynaptic Density (PSD)

A protein-dense region within dendritic spines containing receptors, scaffolding proteins, and signaling molecules.

- Spine Apparatus (SA)

A specialized smooth endoplasmic reticulum (ER)-derived structure within certain dendritic spines; regulates local calcium signaling and plasticity.

- Synaptopodin (SP)

An actin-associated protein essential for spine apparatus formation and synaptic plasticity.

**Calcium Signaling Mechanisms**

- Calcium (Ca^2+^) Signaling

A universal intracellular signaling system regulating synaptic plasticity, gene expression, cytoskeletal remodeling, and neuronal survival.

- Calcium-Induced Calcium Release (CICR)

A process where Ca^2+^ influx triggers further Ca^2+^ release from intracellular stores via ryanodine receptors.

- Store-Operated Calcium Entry (SOCE)

Calcium influx mechanism activated upon ER calcium depletion, mediated by STIM and Orai channels.

- Stromal Interaction Molecules (STIM1/STIM2)

ER calcium sensors that activate SOCE upon calcium depletion.

- Orai Channels (ORAI)

Plasma membrane calcium channels activated by STIM proteins during SOCE.

- Inositol 1,4,5-Trisphosphate Receptors (IP3Rs)

ER membrane receptors that release Ca^2+^ upon IP3 binding.

- Ryanodine Receptors (RyRs)

Calcium release channels located on ER membranes involved in CICR.

- Sarco/Endoplasmic Reticulum Ca^2+^-ATPase (SERCA)

ATP-dependent pump transporting Ca^2+^ back into the ER to restore calcium homeostasis.

- N-Methyl-D-Aspartate Receptor (NMDAR)

A glutamate receptor and calcium-permeable ion channel critical for synaptic plasticity.

**Synaptic Plasticity**

- Long-Term Potentiation (LTP)

Activity-dependent strengthening of synapses associated with spine enlargement and increased receptor density.

- Long-Term Depression (LTD)

Activity-dependent weakening of synapses associated with spine shrinkage and receptor removal.

- Hebbian Plasticity

Synaptic strengthening based on correlated pre- and postsynaptic activity.

**Microtubule Dynamics and Associated Proteins**

- Plus-End Tracking Proteins (+TIPs)

Proteins that bind to growing microtubule plus ends and regulate microtubule dynamics.

- End-Binding Protein 3 (EB3)

A neuron-enriched +TIP that regulates microtubule growth, dendritic spine invasion, and interacts with STIM2.

- End-Binding Protein 1 (EB1)

A +TIP protein involved in microtubule dynamics and tau regulation.

- Tip-Attachment Complex (TAC)

A complex linking ER tubules to growing microtubule plus ends via STIM1–EB1 interaction.

- Axon Initial Segment (AIS)

Specialized proximal axonal region responsible for action potential initiation and cytoskeletal organization.

**Proteases and Calcium-Dependent Enzymes**

- Calpain

A calcium-activated cysteine protease that cleaves cytoskeletal and synaptic proteins under pathological calcium elevation.

- Calpastatin

Endogenous inhibitor of calpain.

- Calcineurin (CaN)

A calcium/calmodulin-dependent phosphatase involved in synaptic signaling and neurodegeneration.

**Pathological Proteins**

- Tau

A microtubule-associated protein that stabilizes microtubules; hyperphosphorylated tau forms neurofibrillary tangles.

- α-Synuclein

A presynaptic protein that aggregates in Parkinson’s disease and disrupts cytoskeletal organization.

- β-Amyloid (Aβ)

A peptide derived from amyloid precursor protein; forms plaques and contributes to calcium dysregulation in AD.

- TDP-43

A DNA/RNA-binding protein implicated in ALS and frontotemporal dementia; undergoes pathological aggregation.

**Therapeutic Strategies**

- Positive Allosteric Modulators (PAMs)

Compounds enhancing protein function without directly activating the primary binding site (e.g., SERCA PAMs).

- TRPC6 Activators

Compounds that stimulate TRPC6 channels to promote synaptic stability and calcium homeostasis.

- Microtubule-Stabilizing Agents

Compounds (e.g., taxane derivatives) that enhance microtubule stability to restore axonal transport.

- ROCK Inhibitors

Inhibitors of Rho-associated kinase that modulate actin cytoskeleton dynamics.

- GSK-3β Inhibitors

Compounds reducing tau phosphorylation and cytoskeletal pathology.

- HDAC6 Inhibitors

Agents that increase microtubule acetylation and stability.

**Emerging Concepts**

- Calcium Hypothesis of Neurodegeneration

Theory proposing that chronic intracellular calcium dysregulation drives neurodegenerative pathology.

- Cytoskeleton–Calcium Interplay

Bidirectional relationship in which calcium regulates cytoskeletal dynamics and cytoskeletal structures influence calcium signaling.

- Prion-like Propagation

Cell-to-cell spread of misfolded proteins via mechanisms such as tunneling nanotubes or extracellular vesicles.

## Data Availability

No new data were created or analyzed in this study. Data sharing is not applicable to this article.
